# The role of antibiotic-derived mycobacterial vesicles in tuberculosis pathogenesis

**DOI:** 10.1038/s41598-024-79215-3

**Published:** 2024-11-15

**Authors:** C. J. Davids, K. Umashankar-Rao, J. Kassaliete, S. Ahmadi, L. Happonen, C. Welinder, C. Tullberg, C. Grey, M. Puthia, Gabriela Godaly

**Affiliations:** 1 Department of Microbiology, Immunology and Glycobiology, Institution of Laboratory Medicine, Lund University, Lund, Sweden; 2 Division of Infection Medicine, Department of Clinical Sciences Lund, Lund University, Lund, Sweden; 3 Swedish National Infrastructure for Biological Mass Spectrometry, BioMS, Lund, Sweden; 4 Division of Biotechnology, Department of Chemistry, Lund University, Lund, Sweden; 5 Department of Dermatology and Venereology, Institution of Clinical Sciences, Lund University, Lund, Sweden

**Keywords:** Antibiotics, Clinical microbiology, Infection

## Abstract

Pulmonary tuberculosis (TB) causes progressive and irreversible damage to lung tissue, a damage that may not fully resolve after treatment. Mycobacterial vesicles (MVs), which are poorly understood, may contribute to TB pathology. This study investigated the effects of stress, such as treatment with conventional TB antibiotics rifampicin, isoniazid, ethambutol, or treatment with an antimycobacterial peptide (NZX), on mycobacterial vesiculation. Stress from minimal inhibitory concentrations of antibiotics, or peptide all increased MV formation. Electron microscopy and lipid profiling revealed that these vesicles, about 40 nm in size, were released from the bacterial inner membrane and consisted of apolar lipids. Using mass spectrometry, the study identified key differences in MVs protein cargo dependent on the antibiotic used, especially with ethambutol-induced MVs that contained proteins from several mycobacterial pathways. Additionally, toxicology analysis using different concentrations of MVs on primary human macrophages and the monocytic cells indicated that MVs from the different treatments were not toxic to human cells, however induced specific inflammatory profiles. In conclusion, this study identified mycobacterial vesicles to be a potential contributor to tuberculosis pathology.

**Supplementary Information:**

The online version contains supplementary material available at 10.1038/s41598-024-79215-3.

## Introduction

Tuberculosis (TB) is caused by the pathogen *Mycobacterium tuberculosis* (Mtb) infecting 10 million new individuals every year. Pulmonary TB is curable with a six-month course treatment standard of four antibiotics. Although successful treatment prevents death, many TB survivors experience long-term health consequences, with increasing evidence of long-term disability and elevated mortality risks. Studies have demonstrated increased pulmonary impairment after successful TB treatment such as chronic obstructive pulmonary disease (COPD), spirometric restriction, pulmonary hypertension, bronchiectasis, and secondary lung infections^1^. A mortality ratio of 2.91 (95% CI 2.21–3.84) was recently reported in a meta-analysis of tuberculosis survivors, compared with individuals without previous TB. This hitherto unquantified post-tuberculosis burden is now advocated to demand more research effort and political attention^2,3^.

One possible explanation of TB pathology could lie in the bacterial surface. The cell envelope is critical for mycobacterial physiology, primarily because of its crucial protection from hostile environments, mechanical resistance of the bacterial cell, transport of solutes and proteins, as well as adhesion to receptors. The trademark of mycobacteria is their unique abundance in lipids, constituting up to 60% of the cell wall composition^4,5^. These lipids include the exceptionally long chain fatty acids (mycolic acids, MA) covalently linked to the cell wall polysaccharide arabinogalacatan (AG), attached to peptidoglycan (PG) and trehalose monomycolates/dimycolates. These lipids have been attributed to many of the biological properties of mycobacteria^5^. Important features comprise the very high resistance of most mycobacterial species to most of the broad-spectrum antibiotics, except for instance streptomycin and rifampicin^6^. Their recognized impermeability to nutrients is up to 100- to 1000-fold than the most resistant Gram-negative bacteria *Escherichia coli* and *Pseudomonas aeruginosa*^7^.

Budding bacterial membrane vesicles (MVs) play a crucial role in mediating intracellular communication and contribute significantly to pathogen survival and infection^8^. For instance, *E. coli* and *P. aeruginosa* employ MVs as vesicles for transporting virulence factors^9^. Mtb released MVs are known to be packed with molecules that modulate the host’s immune response^10^. These Mtb MVs are composed of structurally complex lipids, including trehalose mono- and dimycolate (TMM, TDM), sulfoglycolipids (SLs), lipoarabinomannan (LAM), and phthiocerol dimycocerosate (PDIM)^11^. These lipids are primarily located in the bacterial outer membrane, which serves as the primary source of most MVs, originating through a process of blebbing and pinching. In contrast, MVs can also originate from the inner bacterial membrane layer, where the constituent lipids differ from those in the outer layer. These inner membrane MVs consist of tetra-acylated phospho-myo-inositol dimannosides, Ac2PIM2, phosphatidylinositol mannosides, PIM6, and other AcPIMs^12^. Notably, Mtb MVs also serve to transfer cargo to other bacteria, helping to eliminate intracellular competition and promoting the survival of their own kind, especially in nutrient-deficient or stressed conditions^13^. Previously, we have investigated a cationic host defence peptide for activity against clinical isolates of MDR *M. tuberculosis*, drug resistant *Staphylococcus aureus* (MRSA) and nontuberculous mycobacterial (NTM)^14–17^. This peptide, named NZX, is a derivative from the peptide plectasin^18,19^, and was analysed in several in vitro and in vivo studies^14–17^. In these studies, we observed that peptide treatment induced bubbling on mycobacterial surface^17^. Currently, not much is known about MV production during TB treatment and its potential role in tuberculosis pathogenesis. Thus, the objective of this study was to determine the composition and effects mycobacterial MVs using conventional anti-TB antibiotics and the peptide NZX.

## Results

### TB antibiotic treatment induces vesicle formation

**Fig. 1:**
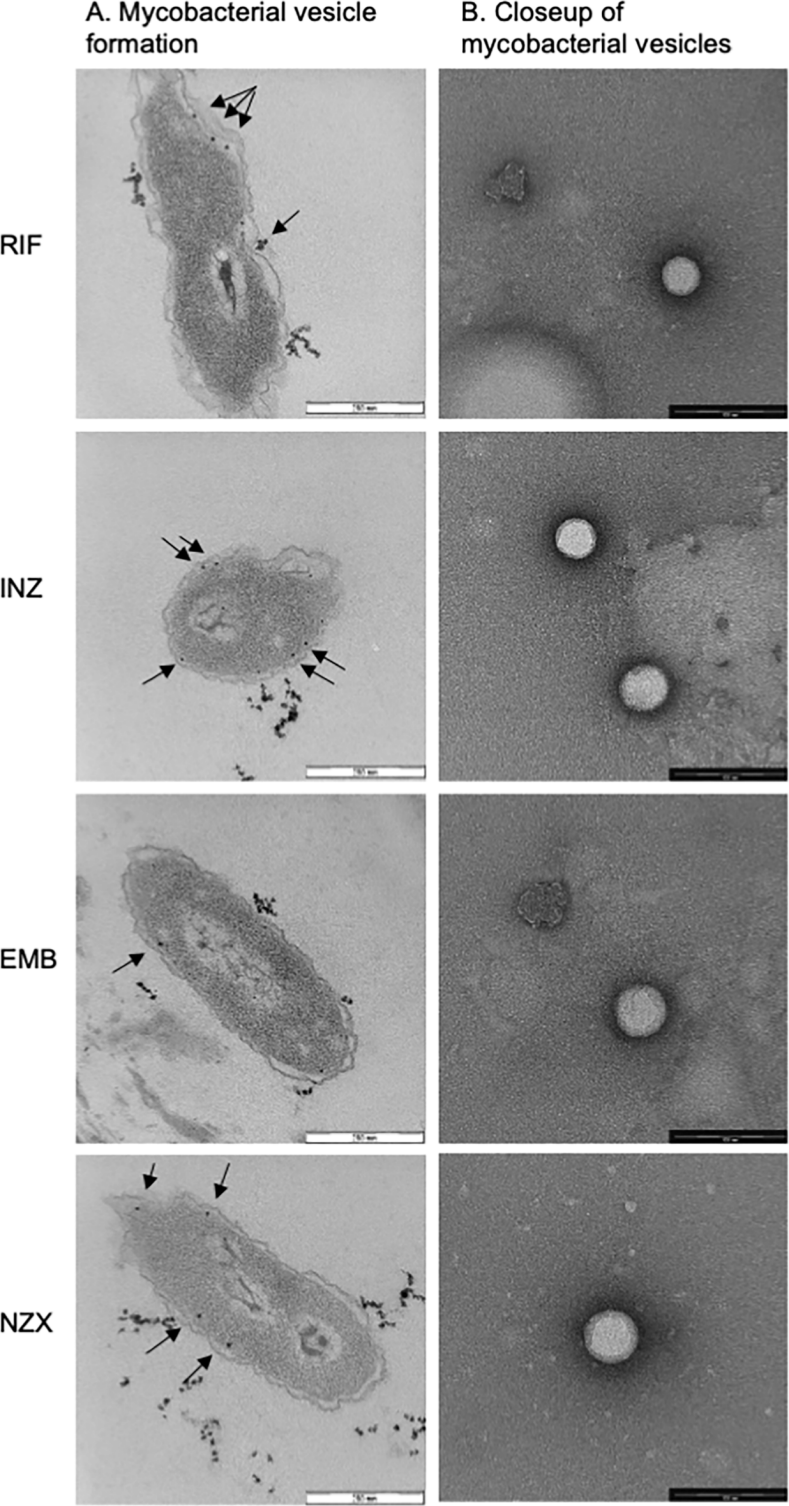
Antibiotic stress induces MVs. (A) EM pictures showing inner membrane vesicle formation (arrows) and aggregation close to the bacteria. (B) Increased magnification showing vesicles approximately 40 nm in diameter and spherical. Some variation in size was observed. Representative images from three analysed batches of vesicles.

Subinhibitory concentrations of antibiotics provide insights into the conditions encountered by bacteria within tissues. The membrane vesicles appeared to originate from the mycobacterial inner membrane, forming clusters as observed through transmission electron microscopy (TEM) (see Fig. 1A). These spherical vesicles exhibited a size range of approximately 40–50 nm and consisting probably of both unilaminar and bilayered lipid membranes (Fig. 1B).

In total, 4–6 batches were isolated from each of the different stimulants. No significant differences in MV production were observed both among different batches and across various treatments, as determined by protein concentration (Fig. 3A). In rifampicin, mycobacterial growth resulted in an average protein of 1058 µg/ml (± 650), isoniazid to 396 µg/ml (± 118), ethambutol to 650 µg/ml (± 252), and NZX to 861 µg/ml (± 262). We were not able to isolate vesicles from antibiotic-untreated mycobacteria.

### Vesicle membrane lipid composition

**Fig. 2:**
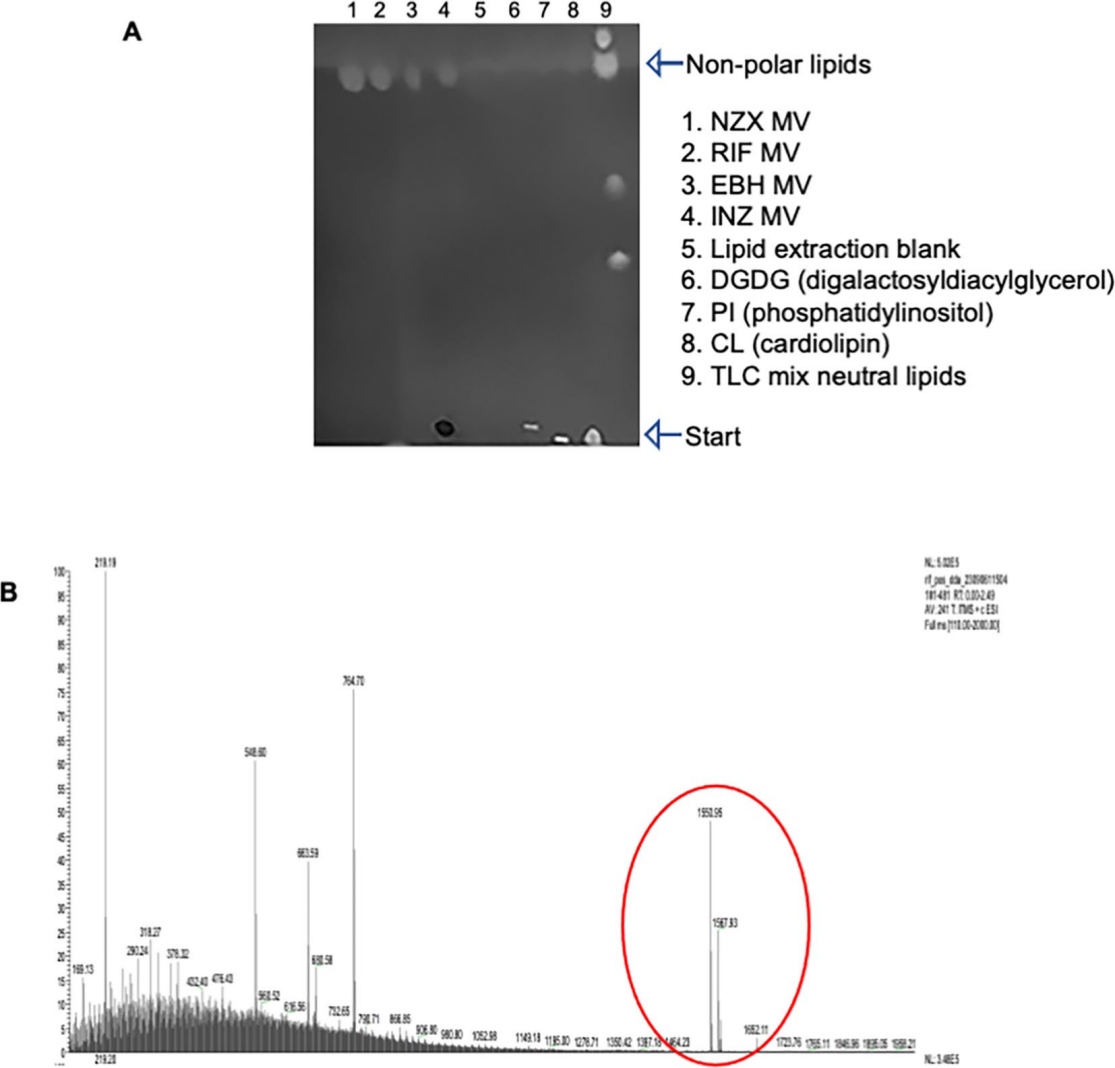
MVs contain nonpolar lipids. (A) TLC separation of lipids showing the non-polar lipids of mycobacterial MVs migrated up the plate. The TLC controls, consisting of polar phospholipids such as phosphatidylinositol (PI), cardiolipin (CL), and digalactosyldiacylglycerol (DGDG) remained stationary at the sample application site on the TLC plate. (B) MS analysis identified apolar trehalose monomycolate (TMM) in mycobacterial MVs from RIF and NZX induced MVs. TMM was not definitively identified in MVs induced by INH or EMB, and no other lipids could be distinctly identified in the MV lipid samples.

The lipid composition of MVs was investigated using a combination of TLC and mass spectrometry (MS). The mycobacterial cell is characterized by two distinct bilayers: an outer membrane (OM) and an inner membrane (IM). The outer membrane is notably enriched with mycolic acids covalently linked to cell-wall arabinogalactan, while the inner membrane is abundant in diacyl phosphatidylinositol dimannoside^10^. In the TLC controls, polar phospholipids such as phosphatidylinositol (PI), cardiolipin (CL), and digalactosyldiacylglycerol (DGDG) remained stationary at the origin of the TLC plate. In contrast, the non-polar lipids of MVs migrated up the plate, as depicted in Fig. 2A. To gain further insights into the lipid composition of MVs, MS analysis revealed the presence of apolar trehalose monomycolate (TMM) in MVs. This was particularly evident in vesicles induced by RIF and NZX treatments, as illustrated in Fig. 2B. However, TMM was not definitively identified in MVs induced by **INH**, and no other lipids could be distinctly identified in the MV lipid samples (Supplemented Fig. 1A, B).

### TB antibiotics induce diverse vesicle protein cargo

**Fig. 3:**
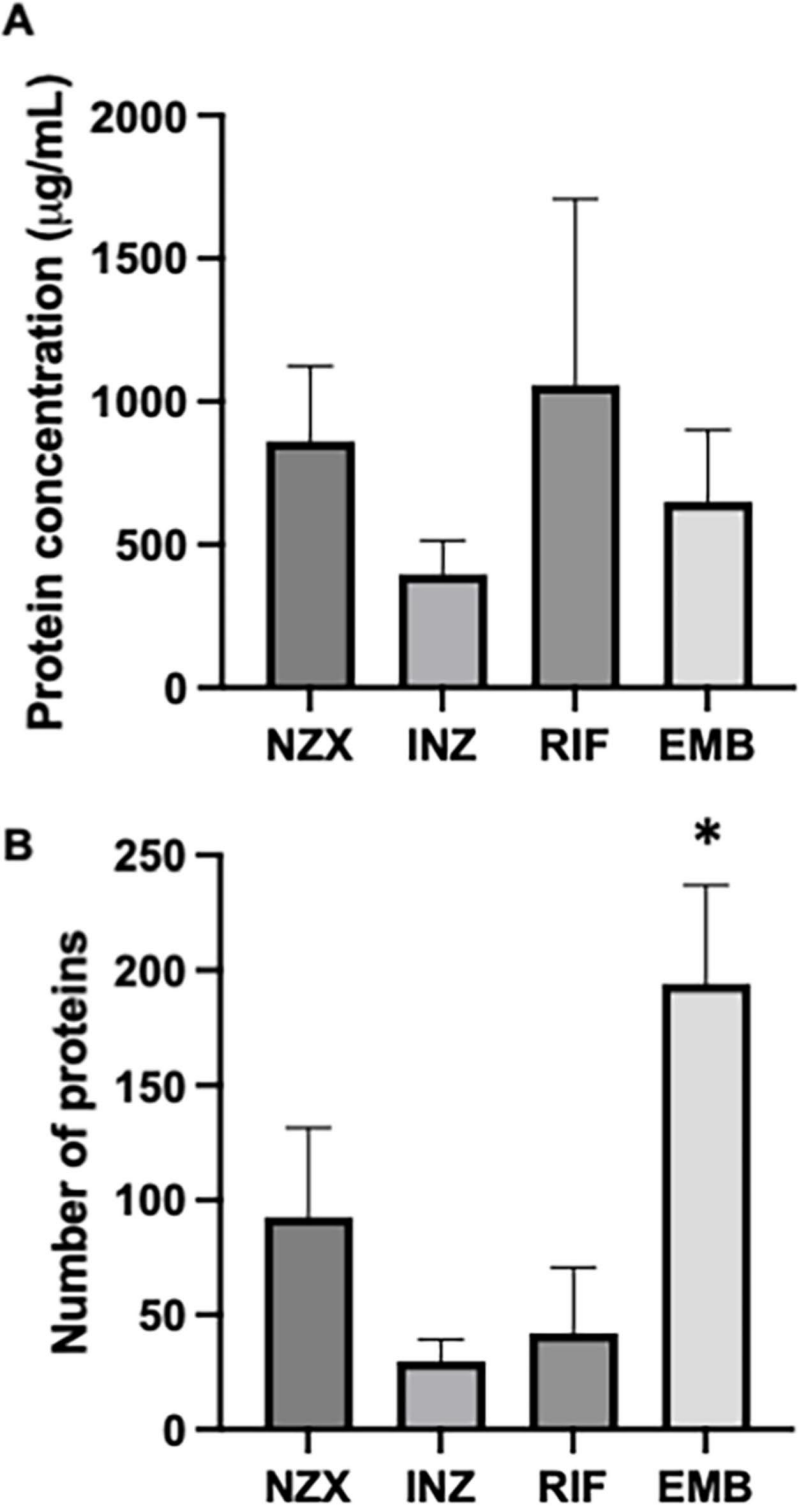
MV protein concentration and variation. (A) Protein concentrations between MV samples were checked prior to analysis and were found to be not significant (*p* < 0.8395). (B) There was a variation in number of proteins with most proteins induced by EMB (*p* < 0.0393).

**Fig. 4:**
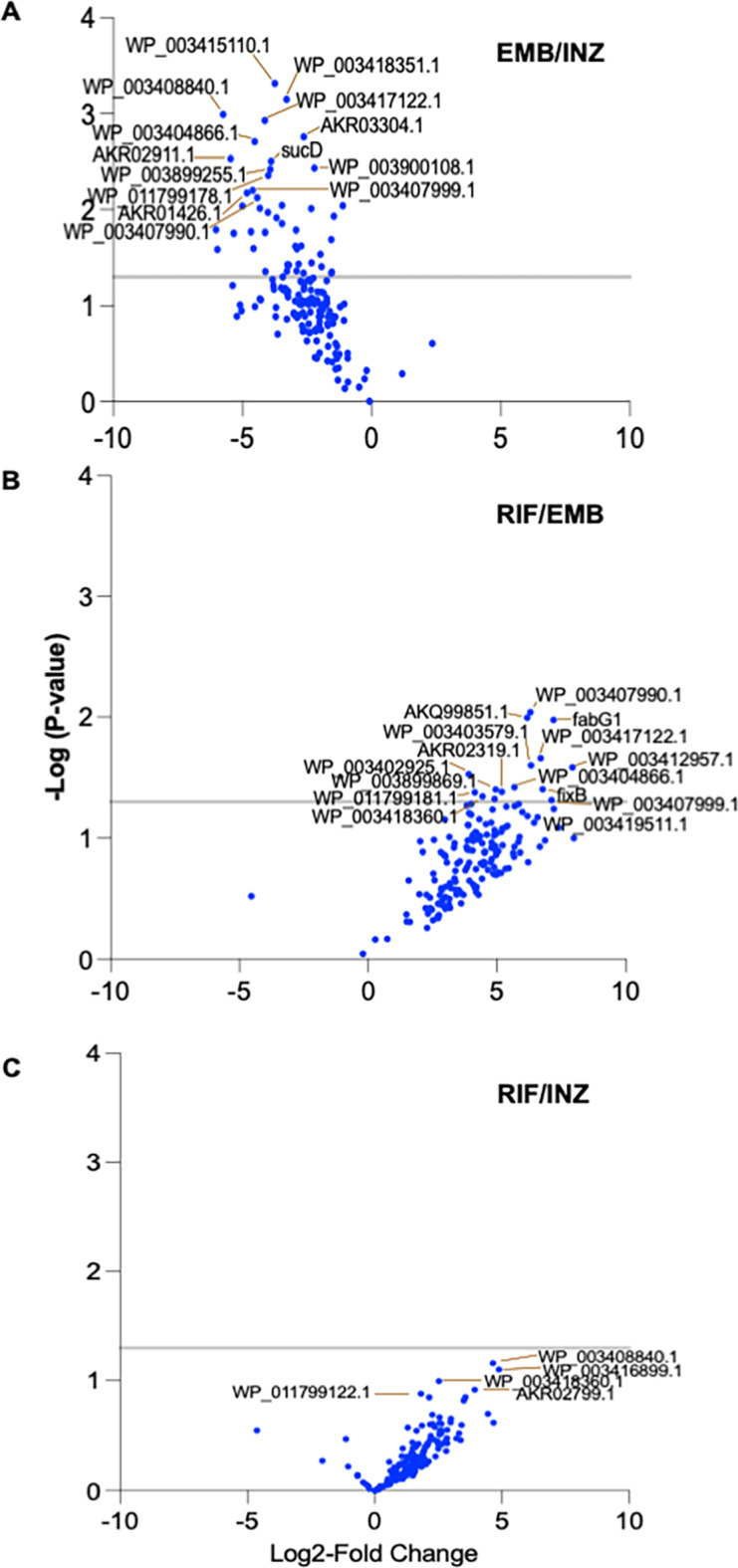

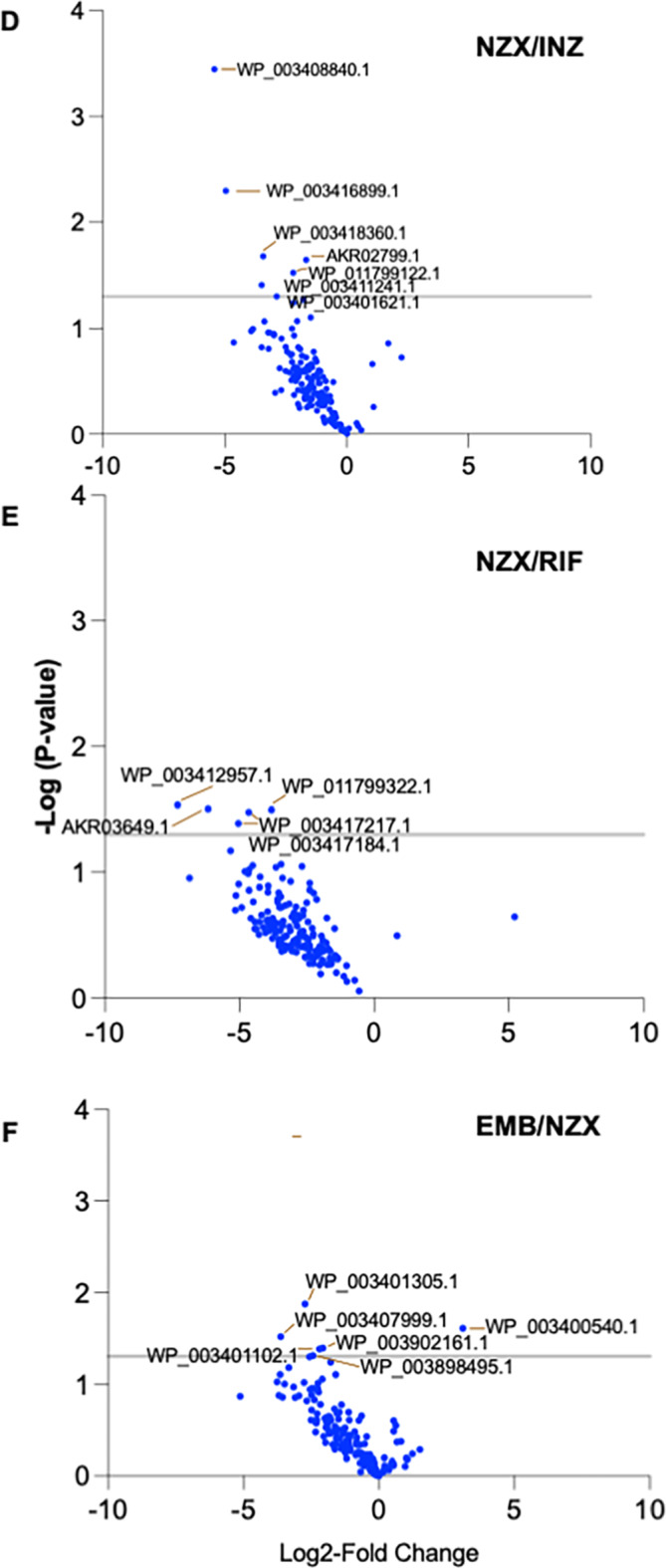
Differences in antibiotic induced MVs protein cargos. Volcano plots identifying significant difference between antibiotic induced MVs proteins. (A) Difference between RIF and EMB, (B) EMB and INH and (C) RIF and INH. Differences between (D) NZX and INH, (B) NZX and RIF and (C) EMB and NZX. Data are presented are from three separate MV batches.

The functions of mycobacterial MVs released into the extracellular environment are determined by the composition of their cargos, such as their protein content. Equal concentrations of antibiotic-induced MVs were loaded onto LC-MS/MS for analysis. The results were subsequently subjected to bioinformatic protein analysis. Protein concentrations were controlled (*p* < 0.9395). Protein analysis revealed that the number of proteins varied, with most proteins induced by EMB (*p* < 0.0393; Fig. 3B). In the identification of protein-protein interaction (PPI) enrichment networks by STRING within the upregulated proteins, differences were observed between EMB-induced MV proteins and those induced by RIF or INH (*p* < 0.05) (Fig. 4A and Supplementary Fig. 2A). In comparison to RIF, EMB-induced MVs were senriched with proteins from the cytoplasmic pathway (Supplemented Fig. 2A). When compared to INH, EMB induced a wide range of MV proteins, including those involved in the fatty acid metabolic process, organic acid metabolic process, carboxylic acid biosynthetic process, and others (see Fig. 4B and Supplementary Fig. 2B).

No significant differences in proteins were observed between RIF and INH (Fig. 4A: C), or between NZX and INH (Fig. 4B: D), or between NZX and RIF (Fig. 4B: E), or between NZX and EMB (Fig. 4B: F).

### Antibiotic-induced mycobacterial MVs induce inflammation and cell death

**Fig. 5:**
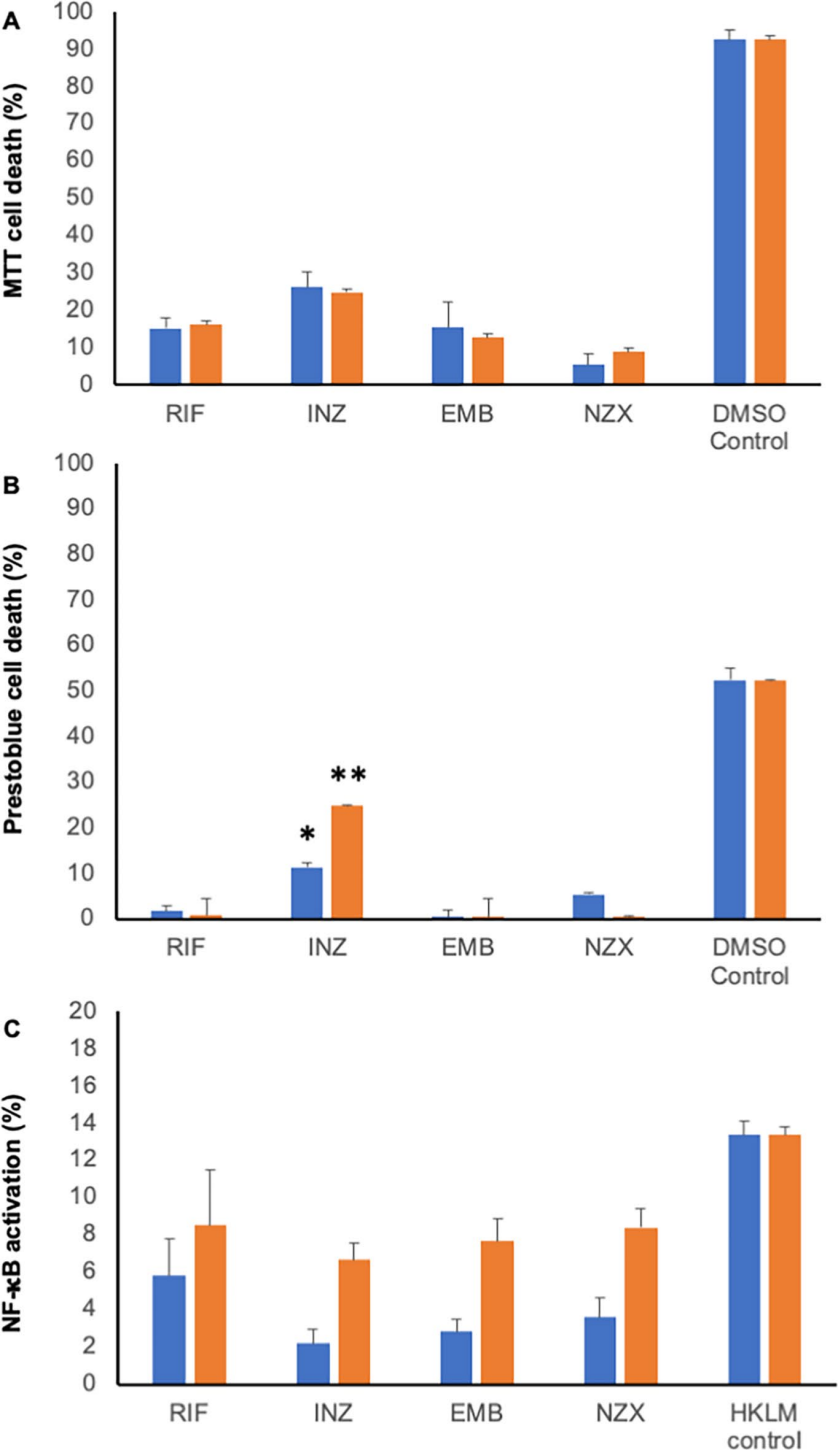
Characteristics of antibiotic induced mycobacterial MVs. Concentration (4 µg/mL (blue) or 40 (orange) µg/mL) related cytotoxicity analysis in primary human macrophages by (A) MTT and in (B) THP-1 cells by Prestoblue (*p* < 0.05 compared to other treatments). (C) Inflammatory response was measure by NF-κB-activation in THP-1 cells after addition of antibiotic induced MVs (4 µg/mL (blue) or 40 (red) µg/mL) or HKLM as control (13.4 (± 3.4 SD) (*n* = 3).

As mycobacterial MVs have previously demonstrated immunomodulatory effects in vivo^20^, we conducted further investigations to assess whether MVs induced by antibiotics exhibit cytotoxic or immunomodulatory effects on human cells. Two different concentrations of MVs, 4 µg/mL (depicted in blue) and 40 µg/mL (depicted in red), were utilized to represent high and low bacterial burdens, as shown in Fig. 5. Generally, higher concentrations of MVs resulted in increased cell death and inflammation. Statistically, MVs derived from isoniazid at both concentrations were found to be more toxic to monocytic cells compared to MVs from other stimulants (*p* = 0.0076 and *p* = 0.0022, respectively; Fig. 5B). The heightened toxicity was also observed in human primary macrophages but was not significant (Fig. 5A). Additionally, an analysis of NF-κB activation following exposure of antibiotic-induced MVs to the monocyte cell line revealed that the MVs at a concentration of 40 µg/mL induced approximately 50% inflammation compared to control (*p* = 0.0144), i.e. heat killed *Listeria monocytogenes* (Fig. 5C).

### Mtb infection attenuates lung fibrosis during TB

**Fig. 6:**
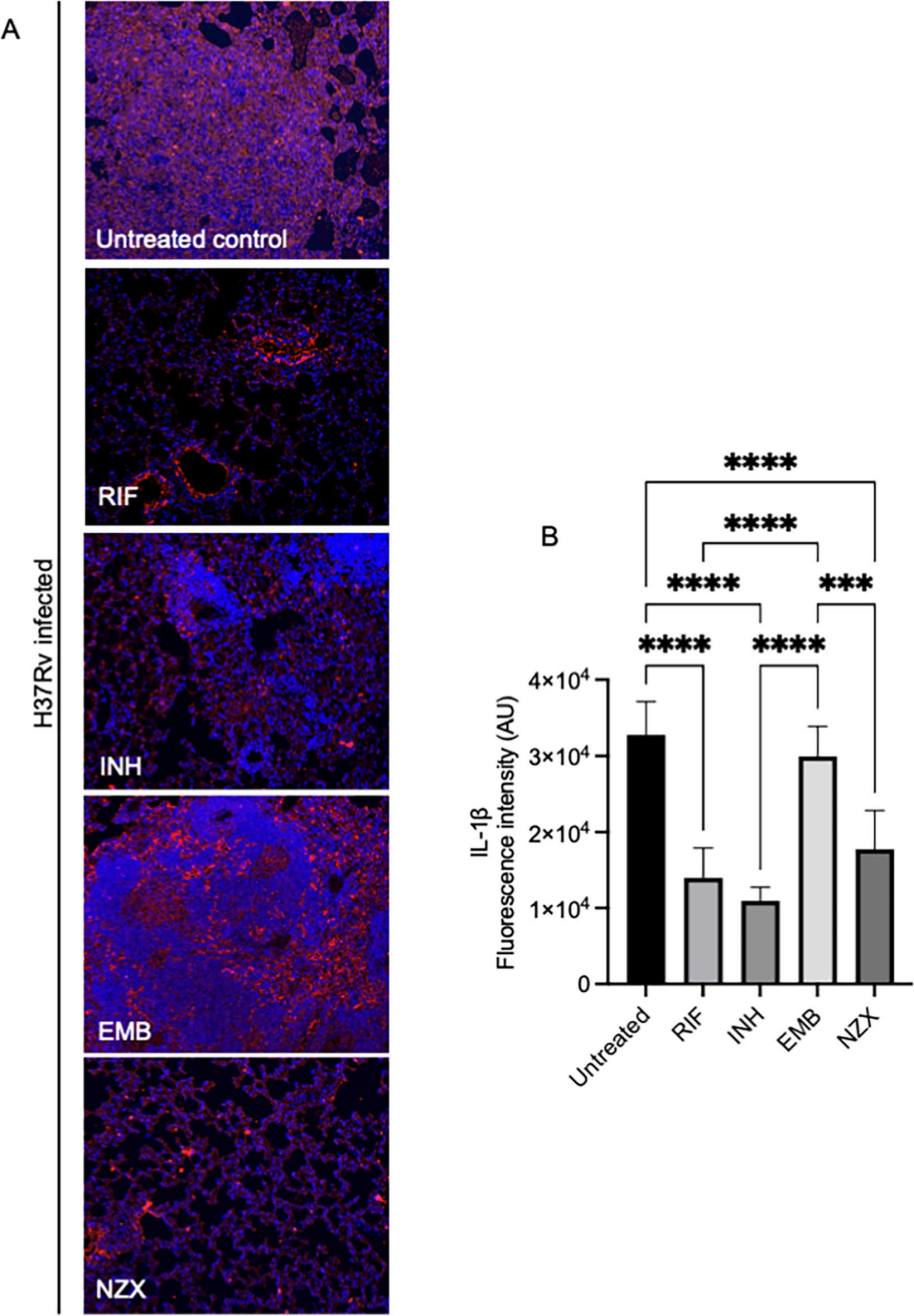
Murine evaluation of TB related lung fibrosis. Immunostaining showing IL-1β expression in lung tissue following TB infection and treatment. Results of the blinded lung inflammatory score. Data are presented as the mean ± SEM (*n* = 4–5).

IL-1β is a potent pro-inflammatory cytokine and linked to lung inflammation and fibrosis^21^. Analysis of lung destruction showed that H37Rv infection in mice severely damaged the lung as analyzed by IL-1β immunostaining. Ethambutol (EMB) treated mice had severe tissue destruction comparable to untreated control. Lung tissue fibrosis was also found in mice treated with rifampicin (RIF), isoniazid (INH), and NZX, but to a lesser degree (Fig. 6A and B).

### Mycobacterial MVs induce selective macrophage cytokine response

**Fig. 7:**
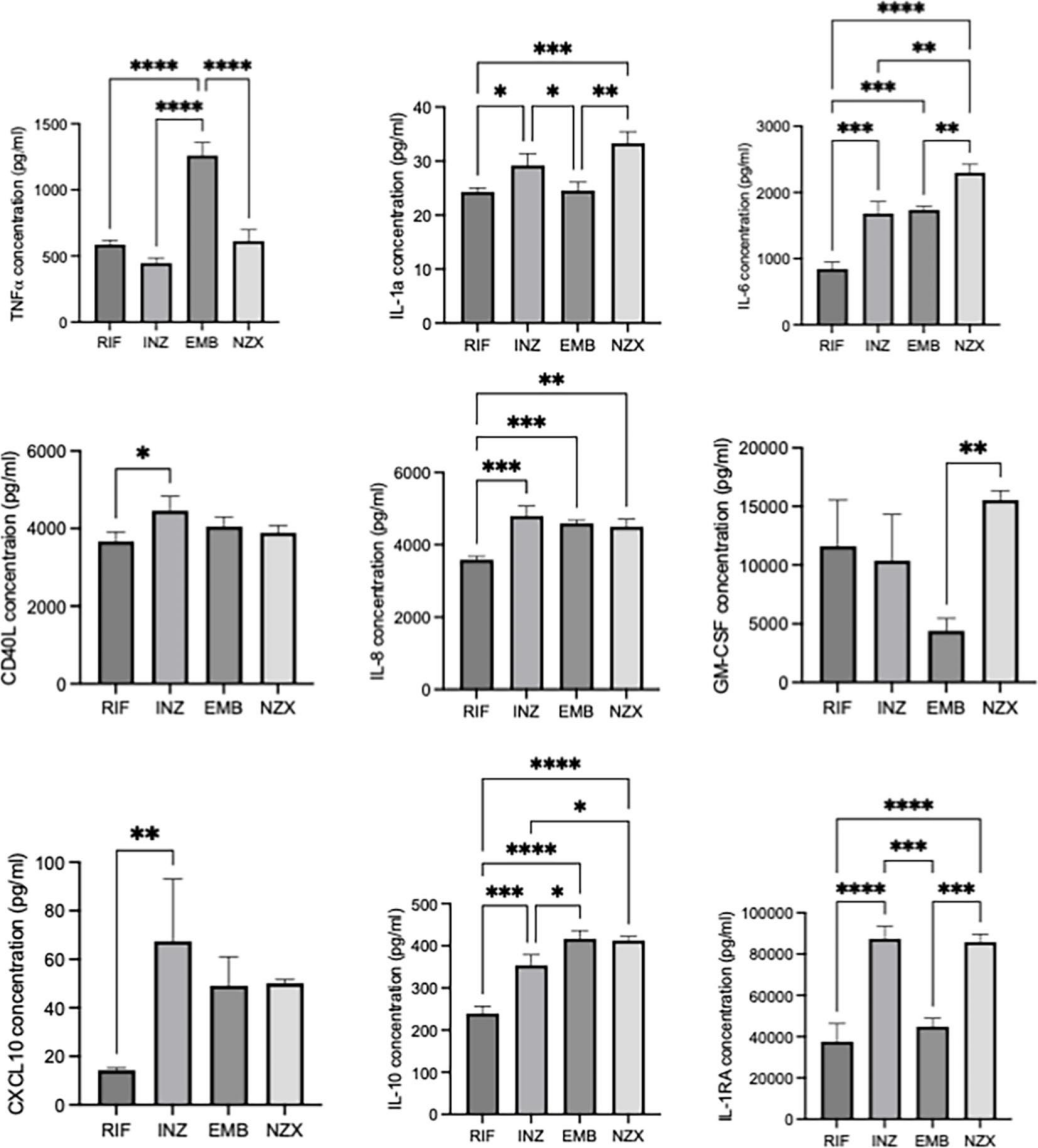
Cytokine profiles from MV stimulated macrophages. Differences in cytokine induction were observed related to MVs from treatment with RIF, INH, EMB and NZX. Both pro-inflammatory and anti-inflammatory responses were induced (*n* = 3).

To evaluate mycobacterial MVs immunomodulatory properties further, we performed a proteomics analysis of cytokines secreted by primary macrophages. Of the 25 investigated cytokines, the MVs stimulated 14, i.e. the pro-inflammatory cytokines TNFα, IL-1α, IL-6, IL-8, inflammatory cytokines CD40L, IL-12, GM-CSF, CCL2, CCL3, CCL4, CXCL10, IL-9, and the anti-inflammatory IL-10 and IL-1RA (Fig. 7). INH stimulated MVs induced high levels of IL-1α, CD40L, IL-8, CXCL10 and IL1RA. EMB stimulated MVs induced highest levels of TNFα and IL-10, while NZX stimulated MVs induced highest levels of IL-1α, IL-6, GM-CSF, IL-10 and IL1RA. There was no significant difference between the antibiotic-induced MVs in cytokines IL-9, IL-12, IL-13, IL-33, CCL2, CCL3, CCL4, Granzyme B and PD-L1 (Supplementary Fig. 2). Vesicle treatment of primary macrophages did not result in secreted IFN-α, IFN-γ, IL-1β, IL-2, IL-4, IL-13, IL-15, or IL-17/IL-17 A.

## Discussion

There is a growing significance in exploring MVs and their cargo of immunologically active molecules that contribute to mycobacterial virulence. We observed an increase in MV production when exposed to conventional TB antibiotics. In our previous study, we reported that peptide-induced bubbling led to cell death in *M. tuberculosis* and was accompanied by a simultaneous increase in MV formation over the exposure time, which sparked our interest in MVs^17^. In this investigation, we noted the bacteria formed vesicles from its inner membrane, aligning with earlier publications on naturally occurring MVs^20^. While the size of the vesicles exhibited some variation, the majority were smaller, approximately 40 nm, spherical and aggregation close to the producing bacterium was observed. Distributed in this way, MVs may act as a form of protective shield, providing sacrificial membrane that enhances resistance against phages or environmental threats. Such threats include antibiotics, which might otherwise directly harm the cell’s envelope^22,23^. Prior research has highlighted that mycobacterial vesicle release is species-dependent, with *M. bovis* BCG and *M. tuberculosis* exhibiting similar production patterns^24^. Enhanced MV production is further known to be caused by stress conditions, such as iron depletion (26). MV production was previously suggested to be regulated by a single genetic element, virR, and by the Pst/SenX3-RegX3 signal transduction pathway^25,26^. MV biogenesis is particularly dependent on dynamin-like proteins, IniA and IniC, that were identified in isoniazid induced MV production^27^. However, the specific regulatory mechanism of MV creation is unclear and needs further study. Our findings indicated comparable MV production across various antibiotic treatments, though potential distinctions in molecular biogenesis, such as the involvement of dynamin-like proteins, remain unclear.

The protein cargo of MVs derived from BCG and *M. tuberculosis* H37Rv revealed enrichment in lipoproteins, for example LprA, LpqH, PstS1 and LppX, compared to the protein cargo in *M. smegmatis*^20^. In addition to variation depending on the mycobacterial strain, it is known that MV protein cargo can be affected by pH and metal content of the culture medium^28,29^. Bacterial membranes are another MV protein source, where the proteins are crucial for the interaction between *M. tuberculosis* and its host^30^. Previous studies report varying numbers of MV proteins, ranging from one to 287, isolated using various techniques^20,26,28,29,31–34^. Lipoproteins were notably abundant, constituting 8% of the MVs’ protein content^34^. We identified 1348 proteins linked to antibiotic induced MVs, with LprO as the only lipoprotein. Significant differences were observed among vesicle proteins induced by different antibiotic treatments, with the highest MV protein cargo induced by EMB treatment. Variances were also noted in antibiotic treatment, affecting the selection of proteins from different pathways. In comparison to RIF, EMB induced MVs with proteins associated with the cytoplasmic pathway. While compared to INH, EMB exhibited differences in proteins involved in fatty acid metabolic processes, organic acid metabolic processes, and carboxylic acid biosynthetic processes. As current studies on MV protein compositions are still insufficient^34^, these intriguing differences in MV antibiotic-induced protein content add further insights to the understanding of mycobacterial vesiculation.

There are many reasons that emphasize the importance of characterizing the composition of lipids in MV interactions, including their interaction with human cells. Previous studies on *M. bovis* BCG MVs identified polar lipids such as cardiolipin, phosphatidylethanolamine (PE), phosphatidylinositol, phosphatidylinositol mannosides (PIMs), as well as the lipoglycan lipoarabinomannan (LAM) and diacylated phosphatidylinositol dimannoside (Ac_2_PIM_2_), suggesting MV formation from the bacterial outer membrane^20^. However, since no mycolic acid esters, the major lipids in the outer membrane of mycobacteria, were detected, the authors concluded that these MVs might originate from the inner membrane^20^. In contrast, Chiplunkar et al. discovered in *M. avium* that MVs contain lipids from the outermost layers of the cell wall^28^. Conversely, our findings reveal that lipids in all antibiotic- and minimal medium-stimulated vesicles were apolar. We also clearly identified apolar trehalose monomycolate (TMM) in RIF and NZX-stimulated MVs. Trehalose plays a crucial role in mycomembrane lipid synthesis in mycobacteria, facilitating the transfer of mycolic acids as trehalose monomycolate (TMM)^35–37^. Through the mycolic acid-exchanging enzyme Ag85, TMM yields key mycomembrane components such as arabinogalactan mycolate (AGM) and trehalose dimycolate (TDM)^38^. This essential pathway is vital for mycobacterial viability and is a target for anti-TB drugs. However, the lipid composition of MVs is still contradictory and requires more studies.

MVs’ containment of membrane proteins and lipids makes them a rich source of possible antigens for priming immune responses. MVs from *M. ulcerans* contain the toxin mycolactone, which has been shown to be toxic to human cells and induces a robust inflammatory response both in vitro and in vivo^39,40^. *M. tuberculosis* MVs contain TLR2 lipoprotein agonists that induce an inflammatory response in macrophages and wild-type mice, but not in TLR2-deficient mice^20,25^. MVs have further been shown to regulate immune responses and disseminate mycobacterial components by inhibiting macrophage and T cell function but promoting MHC class II antigen presentation^41,42^. Variety of methods to quantify MVs in these studies impacts the possibility to compare the results. However, we used two different concentrations of MVs and found that all MVs were moderately toxic to human cells. INH induced the statistically highest cell toxicity at both concentrations but was equally inflammatory as the other antibiotics, as measured by NF-кB activation. The inflammatory induction by antibiotic-isolated MVs was further supported by a broad cytokine response from primary human macrophages. These pro- and anti-inflammatory cytokines have been previously linked to TB pathogenesis, diagnostics, and treatment monitoring^43–46^. A limitation of this study is that the antimycobacterial activity of the antibiotics was evaluated against *M. bovis* BCG, a member of the Mtb complex. Although BCG exhibits 99.9% genetic identity with Mtb, it lacks several genes present in Mtb^47^. However, previous research has shown that Mtb and BCG, but not other mycobacterial strains, secrete extracellular vesicles (EVs) enriched in specific lipoproteins and proteins^20^. Our data suggest that antibiotic treatment-induced MVs could contribute to pulmonary TB-associated pathology.

To summarize, we report herein that conventional treatment with TB antibiotics increases mycobacterial vesicle formation. The formed MVs could impact TB pathology in two ways: by protecting the bacteria^22,23^ and by delivering cargo of mycobacterial lipids and proteins to induce inflammation in human cells. Interestingly, different antibiotics induce different protein cargos, with EMB inducing specific proteins from an array of essential mycobacterial pathways which could explain our previous observation of EMB tissue destruction^16^. This diversity depending on imposed stress sheds further light on the mechanisms of vesiculation. We have thus identified a potential factor in tuberculosis pathogenesis, shedding light on a potential aspect of mycobacterial response toward conventional antibiotics.

## Materials and methods

### Bacteria

For murine TB experiments, we used *M. tuberculosis* H37Rv (a kind gift from Christophe Guilhot, Institut de Pharmacologie et de Biologie Structurale (IPBS), Toulouse, France). For vesicle analysis, *Mycobacterium bovis* bacillus Calmette-Guerin (BCG) Danish (Staten Serum Institute, Copenhagen, Denmark) was prepared as previously described^48^. Briefly, the mycobacteria were grown in Middlebrook 7H9 broth, supplemented with 0.05% Tween 80, 0.2% glycerol and 10% ADC enrichment (Middlebrook Albumin Dextrose Catalase Supplement, Becton Dickinson, Oxford, UK). The culture was washed twice with sterile PBS, re-suspended in broth, and then dispensed into vials. Glycerol was added to a final concentration of 25% and the vials were frozen at − 80 °C. Prior to each experiment, a vial was defrosted, added to 9 ml of 7H9/ADC medium, and incubated with shaking for 72 h at 37 °C. The Mycobacteria was then centrifuged for 10 min at 3000×g, washed twice with PBS, and re-suspended in 10 ml of PBS.

### Cell culture

Human venous blood mononuclear cells were obtained from healthy volunteers according to the manufacturer’s instructions as previously described^15^. In summary, mononuclear cells were isolated from intravenously collected blood using a lymphoprep density gradient system (Axis-Shield, Oslo, Norway) following the manufacturer’s guidelines. Pure monocytes were obtained by applying CD14 microbeads to the cell suspension, washed, and passed through an LS-column, as per the manufacturer’s specifications (Miltenyi Biotec, USA). The monocytes were counted (Sysmex), diluted in RPMI 1640 supplemented with 5% FCS, 1 mM Sodium Pyruvate, 0.1 mg/ml Gentamicin (Gibco, Life Technologies) and 50 ng/ml GM-CSF (R&D systems) and seeded in 96-well plates (40,000 cells per well) for a week to differentiate into macrophages. Infection experiments were conducted in RPMI 1640 without gentamicin.

The human monocyte cell line, THP-1-XBlue™-CD14 (Invivogen, San Diego, USA) was cultured in RPMI 1640 supplemented with 10% FCS, Antibiotic-Antimycocytic, Zeocin, and Geneticin (15240062, R25005, 10131035, Gibco, Life Technologies).

### Transmission electron microscopy

The visualization of vesicle formation and vesicle shape from antibiotic treatment of the mycobacteria, bacteria or isolated vesicles were processed as previously described^49^. Briefly, samples were adsorbed onto carbon-coated copper grids for 2 min, followed by a brief wash with two drops of water. Subsequently, negative staining was performed using two drops of 0.75% uranyl formate. The grids were made hydrophilic through glow discharge at low pressure in the air. All samples were meticulously examined using a Jeol JEM 1230 electron microscope operating at an 80 kV accelerating voltage. High-quality images were captured using a Gatan Multiscan 791 charge-coupled device camera.

### Isolation of mycobacterial vesicles

MV’s were isolated according to our previous protocol^50^. Briefly, an inoculum culture BCG was grown in 7H9 Middlebrook broth supplemented with 10% ADC (BD Bioscience), 0.05% Tween 80 (Sigma-Aldrich), 0.2% glycerol (ThermoFisher) and incubated at 37 °C. Once the culture reached the mid-exponential phase it was transferred to media containing subinhibitory concentrations (one log_10_ decrease compared to control) of rifampicin (RIF, 0.25 µg/mL), isoniazid (INH, 0.025 µg/mL), ethambutol (EMB, 1.25 µg/mL), NZX (6.07 µg/mL) or no antibiotics^14,51,52^. The bacteria was grown in a 1 L flask until the late exponential phase. The MV’s were harvested by centrifuging the sample at 4000 rpm for 15 min. The pellet was collected in a sterile container and vacuum filtered using a 0.45 μm filter. The remaining supernatant was centrifuged at 4000 rpm for 15 min using 100 kDa exclusion filter tubes. The retentate was rinsed and collected separately. Untreated mycobacteria.

### Vesicle purification

The processing and isolation of MVs were performed according to a previous protocol^20^ along with the incorporation of minor adaptions to these studies. The BCG culture that was grown in the retentate was centrifuged at 4000 rpm followed by 15,000 rpm for 15 min at 4 °C and the supernatant was collected. The supernatant was pelleted at 35,000 rpm for 2 h and resuspended in 1 mL DPBS. The vesicles were further purified using a density-gradient system with Optiprep and centrifuged at 35,000 rpm for 20 h in a swing bucket rotor. Following centrifugation, the band was collected and quantified using a BCA protein assay. The sample was aliquoted into tubes and stored at – 80 °C until further analysis.

### Protein mass spectrometry acquisition

The MV samples were reduced with dithiothreitol to a final concentration of 10 mM and heated at 56 °C for 30 min followed by alkylation with iodoacetamide to a final of 20 mM for 30 min at room temperature in dark. Digestion was performed by adding trypsin in a ratio of 1:50 (Sequencing Grade Modified Trypsin, Part No. V511A, Promega) to the samples and incubated overnight at 37 °C. The digestion was stopped by 20 µL 10% trifluoroacetic acid. The peptides were Speed Vac to dryness and resolved in 2% ACN/0.1% TFA.

The samples were analyzed on an Orbitrap Eclipse Tribrid mass spectrometer (Thermo Fischer Scientific) coupled with an Ultimate 3000 RSLCnano system (Thermo Fischer Scientific). Two-column setup was used on the HPLC system and peptides were loaded into an Acclaim PepMap 100 C18 precolumn (75 μm x 2 cm, Thermo Scientific, Waltham, MA) and then separated on an EASY spray column (75 μm × 25 cm, nanoViper, C18, 2 μm, 100 Å) with the flow rate 300 nL/min. The column temperature was set 45 °C. Solvent A (0.1% FA in water) and solvent B (0.1% FA in 80% ACN) were used to create a nonlinear gradient to elute the peptides. For the gradient, the percentage of solvent B was maintained at 2% for 4 min, increased from 2 to 25% for 100 min and then increased to 40% for 20 min and then increased to 95% for 1 min and then kept at 95% for another 5 min to wash the column.

The samples were analyzed with the positive data-dependent acquisition (DDA) mode. The full MS resolution was set to 120,000 at normal mass range and the AGC target was set to standard with the maximum injection time to auto. The full mass range was set 350–1400 m/z. Precursors were isolated with the isolation window of 1.6 m/z and fragmented by HCD with the normalized collision energy of 30. MS2 was detected in the Orbitrap with the resolution of 15,000. The AGC target and the maximum injection time were set standard and 50 ms, respectively.

Raw DDA data were analyzed using Proteome Discoverer™ 2.5 Software (Thermo Fisher Scientific). Peptide identification employed SEQUEST HT and Mascot against the UniProtKB Mycobacterium strain BCG/Pasteur 1173P2 database (UP000001472). The search parameters included static modification (cysteine carbamidomethylation) and dynamic modifications (N-terminal acetylation). Precursor tolerance was set to 10 ppm, and fragment tolerance was set to 0.02 ppm. Up to 2 missed cleavages were allowed, and Percolator was employed for peptide validation at a maximum q-value of 0.05. Extracted peptides were used for identification and quantification through label-free relative quantification. Chromatographic intensities were extracted and utilized for comparing protein abundance across samples.

### Bioinformatic protein analysis

Raw MS data were analyzed with MaxQuant (v2.4.9.0). The acquired spectra were analysed against the *M. bovis* (strain BCG/Pasteur 1173P2) reference proteome (UniProt proteome ID UP000001472) containing 3891 sequence entries. False discovery rate was set to 1% for both peptides (minimum length of 7 amino acids) and proteins. Fully tryptic digestion was used allowing 2 missed cleavages. Carbamidomethylation (C) was set to static and oxidation (M) as well as protein N-terminal acetylation to variable modifications, respectively. Initial search peptide mass tolerance was set to 20 ppm, and the main search peptide mass tolerance was set to 4.5 ppm. LFQ was selected for label-free quantification, with the minimal LFQ ratio count set at 2. Proteins identified by one peptide only were omitted prior to further data analysis. The LFQ data was analyzed in Perseus (v2.0.9.0). LFQ intensity values were normalized by log2 transformation and mean subtraction. Proteins with less than 25% of valid values (i.e., 3 of 12 samples compared) were filtered out. The remaining missing values were imputed from a Gaussian distribution; width = 0.2 and downshift = 1.8). For volcano plots, differentially enriched proteins in the groups compared were identified by two-sample t test (FDR < 0.05, s0 = 0.1) and visualized by Prism 10 (Graph Pad). The STRING database v12 (http://string-db.org/) was used to predict the potential interactions as well as functional association between vesicle proteins. The combined score of medium confidence > 0.4 was used as the cut-off value in the STRING database.

### Vesicle lipid composition

The lipid investigation was performed according to a previous study^53^. Briefly, the lipids were extracted using chloroform: methanol (1:2 v/v, with 0.05% BHT w/v), followed by the addition of 1 mL 0.15 M acetic acid, 1.25 mL chloroform and 1.25 mL water. Next, the sample was centrifuged at 2500 rpm for 6 min and the lower phase was collected and transferred to separate tubes. The chloroform phase was recovered and dried using N_2_-gas for approximately 30 min. Next, chloroform: methanol (9:1) was added to the individual tubes and used for TLC and LC-MS analysis.

For the thin layer chromatography, the individual samples were applied to a high-performance TLC glass plate (HPTLC; Silica gel 60, Merck) using the ATS4 autosampler (CAMAG) and using methanol as the washing solvent. As TLC controls, we used the TLC Mix 34 for neutral lipid standards (Larodan, Sweden), including monoolein, diolein, triolein, methyl oleate and oleic acid, and the polar phospholipid phosphatidylinositol (PI, Sigma), cardiolipin (CL, Sigma), and digalactosyldiacylglycerol (DGDG, Larodan). The used lipid standard in TLC consisted of specific neutral polar and nonpolar lipids to provide first identification of the isolated vesicle lipids. Next, the TLC plate was left in the development chamber with chloroform: methanol: water (100:14:0.8,v/v/v) as the mobile phase. Once fully developed, the spots were visualized using 0.05% primulin in acetone: water (8.2v/v) or 0.1% 2,7-dichloroflourescein in 99.5% ethanol. The plate was scanned (TLC Scanner 3, CAMAG) and visualized under UV light. Identification of lipid classes was performed by comparing them with lipid standards.

The LC-MS/MS on MVs lipids was performed according to^54^. The MV lipids dissolved in chloroform: methanol 9:1 were diluted 1:10 with ACN (0.1% formic acid) and analysed using a linear ion trap mass spectrometer (LTQ-Velos Pro, Thermo Scientific) connected to a HPLC system (Ultimate 3000, Thermo Scientific) running isocratically at 50 µL/min (1:1 ACN: MQ with 5 mM ammonium formate). Samples were introduced to the MS via a Hamilton syringe at a flow rate of 20 µL/min, and the MS was operated in heated-electrospray ionization (H-ESI)-negative and positive mode with a capillary temperature of 275 °C, no source heating, and source voltage of 3.5 kV. Full-scan was acquired in the range of m/z 110 or 150–2000. Data was collected using data-dependent acquisition performing full-scan experiments and untargeted MS/MS using collisional induced dissociation (CID) fragmentation. The different lipid classes and the acyl-chain composition within each class were identified by their exact mass and characteristic fragmentation of the hydrocarbon chains and headgroups as evidence. LTQ Tune Plus V2.8 and XCalibur V4.4 software were used for MS control, data acquisition and analysis.

### Cytotoxicity assays

Toxicity experiments utilizing human-derived macrophages were conducted in accordance with our prior studies^15^. For this study, primary macrophages were treated with medium containing 4 or 40 µM vesicles and incubated overnight at 37 °C with 5% CO_2_. For cytotoxicity measurement, 10 µl 3-(4,5-dimethylthiazol-2-yl)-2,5 diphenyltetrazolium bromide (MTT) solution (Sigma) was added to each well, and the plate was incubated for another hour at 37 °C with 5% CO₂. The MTT and media mixture was then discarded and 100ul of DMSO was added to dissolve the formazan crystals and place on the shaker for 30 min following the manufacturer’s instructions. The absorbance was analyzed using a spectrophotometer the ELISA plate reader at 535 nm.

Vesicle cytotoxicity was further examined by PrestoBlue^®^ assays. Primary macrophages were treated with 4 or 40 µM vesicles or 50 µM positive control (S-4400, Sigma) for 24 h. Cell viability was assessed with PrestoBlue^®^ fluorescence (A13261, Thermo Scientific) compared to untreated controls, according to the manufacturer’s instructions.

The Human THP-1-XBlue™-CD14 monocytic cell line was stimulated with 4 or 40 µM vesicles or heat-killed *Listeria monocytogenes* (HKLM) as positive control. QUANTI-Blue™ assay was performed according to manufacturer’s instructions (Invivogen, San Diego, USA). Briefly, supernatants from the cells were added to QUANTI-Blue™ substrate for one hour and absorbance was measured at 620 nm. In parallel, the ATPlite™ assay was performed to ensure that the activation was not due to toxicity.

### Inflammatory responses

NF-κB activation was measured in human THP-1-XBlue™-CD14 monocytes following the addition of 4 or 40 µM vesicles or heat-killed *Listeria monocytogenes* (HKLM) as a positive control. Human macrophages were stimulated with 4 or 40 µM vesicles to better depict the possible situation during TB infection. The vesicle stimulated cells were incubated at 37 °C, 5% CO_2_ for up to 24 h. The vesicle-induced samples were stored at – 80 °C until further use.

### Cytokine microarray

The concentration of cytokines from vesicle-induced primary human macrophages were analyzed utilizing the Human Immunotherapy Luminex Performance Assay 25-plex Fixed Panel (CCL2/MCP-1, CCL3/MIP-1α, CCL4/MIP-1β, CD40 Ligand, CXCL10/IP-10, GM-CSF, Granzyme B, IFN-α, IFN-γ, IL-1α, IL-1β, IL-1RA, IL-2, IL-4, IL-6, IL-8/CXCL8, IL-9, IL-10, IL-12 p70, IL-13, IL-15, IL-17/IL-17 A, IL-33, PD-L1/B7-H1, and TNF-α) (R&D Systems). The assays were executed in accordance with the manufacturer’s instructions. Fluorescence intensities of a minimum of 100 beads/analytes were recorded using a Luminex 200 analyzer equipped with xPonent software build 3.1.871.0 (Luminex Corporation), adequately calibrated based on the manufacturer’s instructions. Concentration values were calculated and analyzed using the Prism 10 statistical program, relying on the standard curves as reference.

### Immunohistochemistry

The lung tissue was re-used from a previously performed murine TB infection model^16^. Briefly, six- to eight-week-old female BALB/c mice (Charles River Ltd, UK) were infected with ~ 7 × 10^3^ CFU/ml of *M. tuberculosis* H37Rv via the intranasal route under gaseous anaesthesia with sevoflurane (5 mice in each group). Following 28 days of infection, groups of mice were treated 3 times per week during for 4 weeks with first-line drugs TB-drugs rifampicin (20 mg/kg), isoniazid (25 mg/kg), ethambutol (100 mg/kg) or the peptide NZX (33 mg/kg) diluted in 50 µl PBS by intranasal administration. The control group received 35 µl PBS by the same route. After treatment, the mice were euthanized by receipt of an overdose of the anesthetic sevoflurane, and the lungs were aseptically removed for histology.

Fixed lung tissue was embedded in paraffin blocks and sectioned. Sections were deparaffinized in xylene (5 min, 3 times). To remove xylene, sections were placed in ethanol (5 min in 100% ethanol, 5 min in 95% ethanol, and 5 min in 70% ethanol). Finally, sections were placed in tap water (10 min). Antigen retrieval was performed by placing sections in citrate buffer (95 °C for 20 min). After antigen retrieval, sections were cooled to room temperature and blocked with 1% BSA (Sigma) for 1 h at room temperature. Sections were then stained with primary antibodies (rabbit monoclonal anti-IL-1β antibody, 1:100 dilution, GTX636887, GeneTex) at 4 °C overnight. Sections were washed in PBS (5 min, 3 times) and incubated with secondary antibody (Alexa 568 anti-rabbit IgG, 1:400 dilution, Invitrogen) for 1 h at room temperature. Sections were washed in PBS (5 min, 3 times) and counterstained with DAPI solution (0.05 mM, Sigma-Aldrich). Sections were washed in PBS (5 min, once) and mounted using aqueous mounting medium (Permafluor, ThermoScientific). Overall severity score was calculated for each animal by adding the individual scores. Scores for animals from each experimental group were pooled and averaged. Imaging was performed by fluorescence microscopy and obtained images were analyzed using ImageJ software. Five high-power fields (×200) were randomly selected and the intensity of IL-1β staining was quantified.

### Statistical analysis

Graphs and statistics were generated using the Prism software (version 10). Significance, where indicated, was calculated using ordinary one-way ANOVA with Tukey’s multiple comparisons. Significance was accepted at *p < 0.05, **p < 0.01, or ***p < 0.001.

### Ethics statement

This study is reported in accordance with ARRIVE guidelines (https://arriveguidelines.org). All animal procedures were performed under the license issued by the UK Home Office and in accordance with guidelines and regulations as stated at the Animal Scientific Procedures Act of 1986. The animal studies have been approved by the Local Animal Welfare and Ethical Review Board (London, UK) (Numbers PPL 70/7160 and 70/8653). For cytotoxicity monocyte isolation the blood was donated by healthy volunteers. Verbal and written informed consent was obtained from all participants. The study was approved by Regional Ethical Review Board and performed in accordance with relevant guidelines and regulations (Dnr 2011/403 and 2014/35).

## Electronic supplementary material

Below is the link to the electronic supplementary material.

Supplementary Material 1

## Data Availability

The raw data are available from the corresponding author, Prof. Gabriela Godaly, upon request (Email: gabriela.godaly@med.lu.se).
