# Supplementary material for: The role of antibiotic-derived mycobacterial vesicles in tuberculosis pathogenesis
Source: Sci Rep. 2024 Nov 15;14:28198. doi: 10.1038/s41598-024-79215-3 (PMC11568285; doi:10.1038/s41598-024-79215-3)
Supplement: Supplementary file 1 — Supplementary Material 1 [file 41598_2024_79215_MOESM1_ESM.pdf]

The role of antibiotic-derived mycobacterial vesicles in tuberculosis pathogenesis

Davids CJ[1], Umashankar-Rao K[1], Kassaliete J[1], Ahmadi S[1], Happonen L[2], Welinder C[3], Tullberg C[4], Grey C[4], Puthia M[5], Godaly G[1].

[1]Department of Microbiology, Immunology and Glycobiology, Institution of Laboratory Medicine, Lund University, Lund, Sweden. [2]Department of Clinical Sciences Lund, Division of Infection Medicine, Lund University, Lund, Sweden. [3]Swedish National Infrastructure for Biological Mass Spectrometry, BioMS, Lund, Sweden. [4]Division of Biotechnology, Department of Chemistry, Lund University, Lund, Sweden. [5]Department of Dermatology and Venereology, Institution of Clinical Sciences, Lund University, Lund, Sweden.

Supplementary Figure 1A

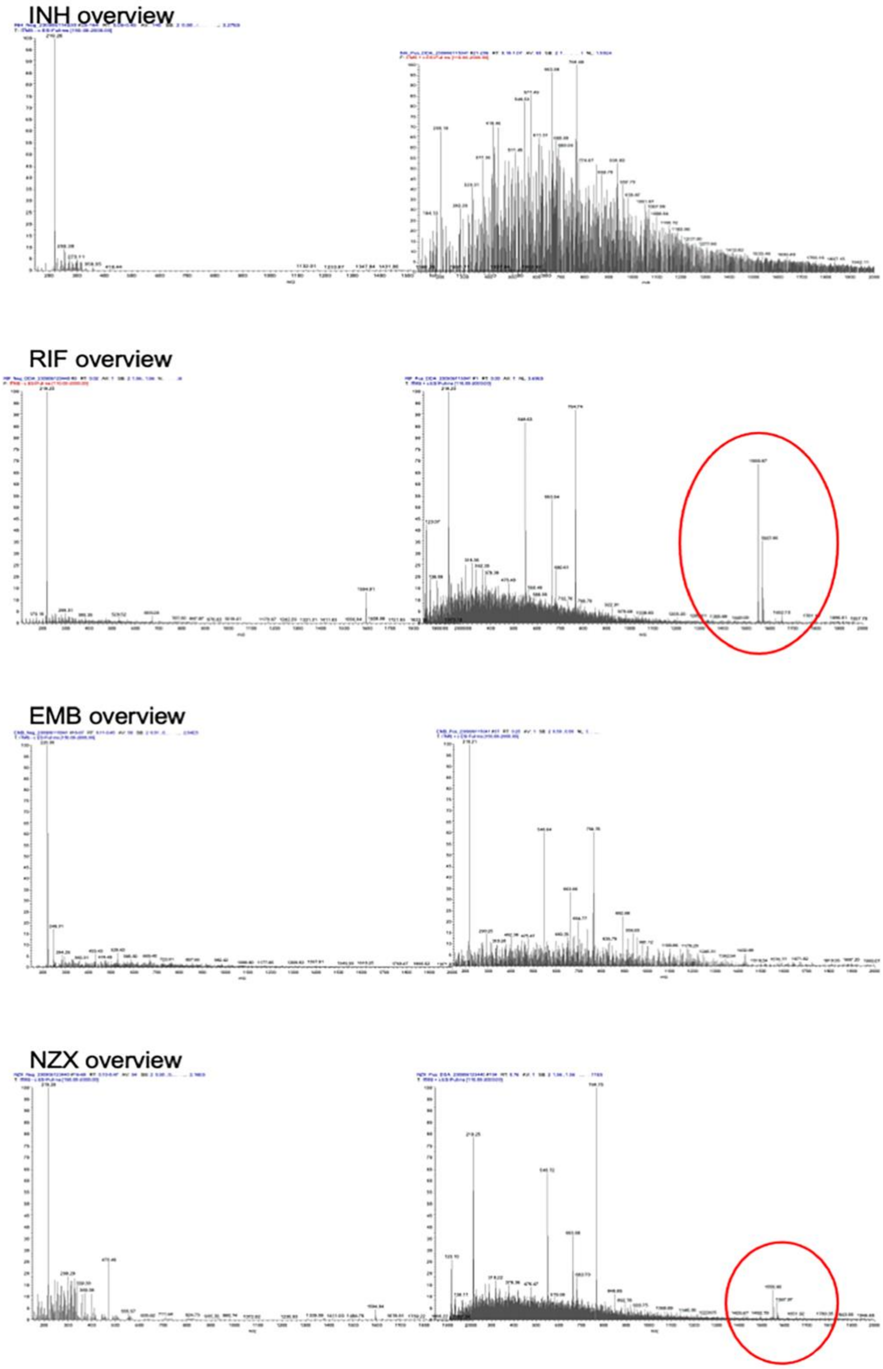

Supplemented Figure 1. Label-free quantification (LFQ) of complete MV protein acquisition. MS analysis identified apolar trehalose monomycolate (TMM) in mycobacterial MVs from RIF and NZX induced MVs (A). TMM was not definitively identified in MVs induced by INH or EMB, and no other lipids could be distinctly identified in the MV lipid samples (B).

Supplementary Figure 1B

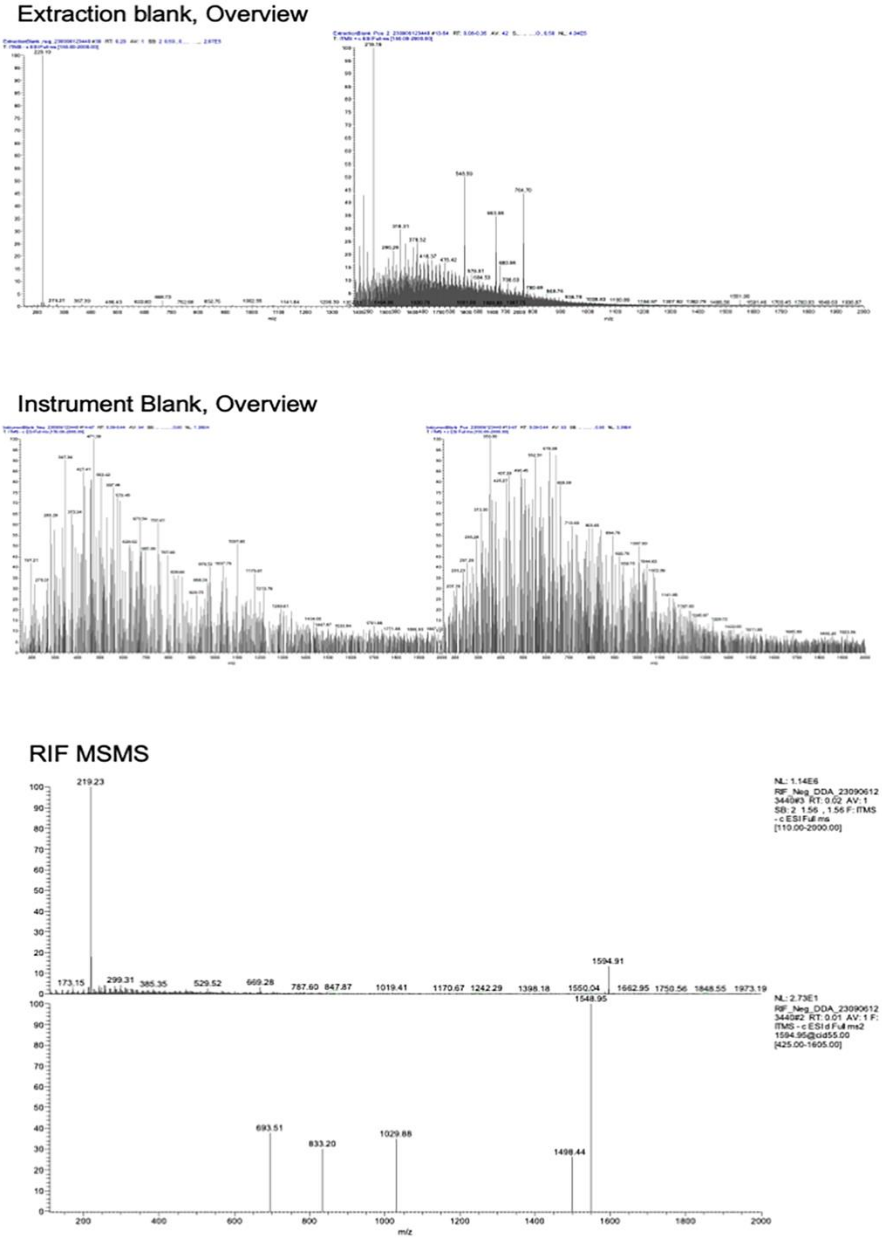

Supplemented Figure 1. Label-free quantification (LFQ) of complete MV protein acquisition. MS analysis identified apolar trehalose monomycolate (TMM) in mycobacterial MVs from RIF and NZX induced MVs (A). TMM was not definitively identified in MVs induced by INH or EMB, and no other lipids could be distinctly identified in the MV lipid samples (B).

Supplementary Figure 2A

A

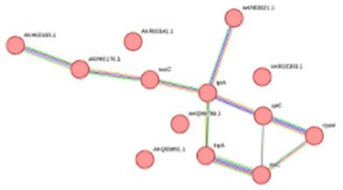

RIF/EMB

PPI enrichment p-value: 0.0238			
**GO-term**	**Description**	**Strength**	**FDR**
GOCC:0005737	Cytoplasm	0.49	0.0094

B

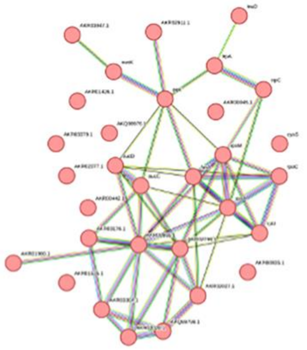

EMB/INZ

PPI enrichment p-value: 0.0173			
**GO-term***	**Description**	**Strength**	**FDR**
GO:0006631	Fatty acid metabolic process	0.73	0.0169
GO:0046394	Carboxylic acid biosynthetic process	0.7	0.0080

* Table showing only the top enrichment analysis result

C

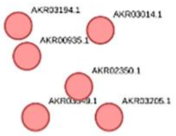

NZX/RIF

PPI enrichment p-value: 1			
**Term ID**	**Description**	**Strength**	**FDR**
GO:0016998	Cell wall macromolecule catabolic process	2.64	0.0268

Supplemented Figure 2. Identification of protein–protein interaction (PPI) enrichment networks of the significantly upregulated proteins in antibiotic induced MVs (red colour). Differences in MV protein content between (A) RIF and EMB, (B) EMB and INZ, (C) RIF and INZ, (D) NZX and RIF, (E) NZX and INZ, (F) EMB and NZX. Tables showing PPI p values, GO term IDs and their FDR values.

Supplementary Figure 2B

D

RIF/INZ

PPI enrichment p-value: 1
No significant enrichment detected

E

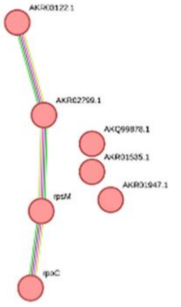

NZX/INZ

PPI enrichment p-value: 0.322	
No significant enrichment detected	

F

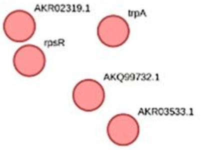

EMB/NZX

PPI enrichment p-value: 1	
No significant enrichment detected	

Supplemented Figure 2. Identification of protein–protein interaction (PPI) enrichment networks of the significantly upregulated proteins in antibiotic induced MVs (red colour). Differences in MV protein content between (A) RIF and EMB, (B) EMB and INZ, (C) RIF and INZ, (D) NZX and RIF, (E) NZX and INZ, (F) EMB and NZX. Tables showing PPI p values, GO term IDs and their FDR values.

Supplementary Figure 3

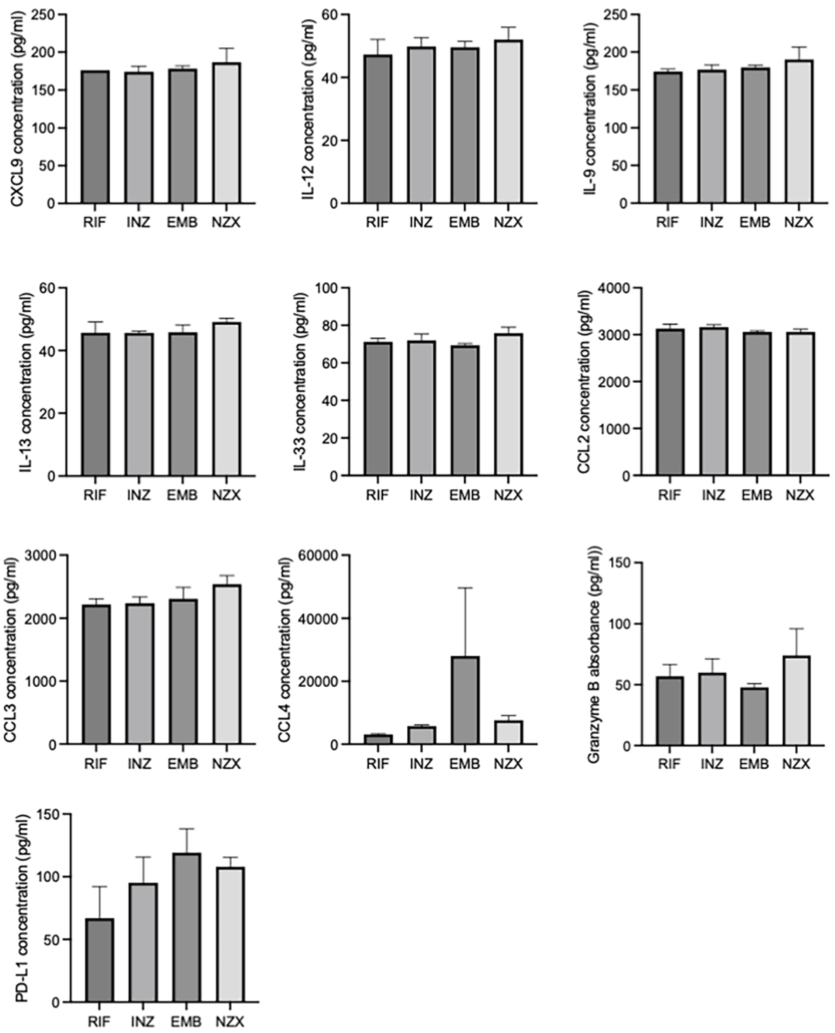

Supplemented Figure 3. Macrophage cytokine from antibiotic induced MV**.** Antibiotic treatment induced MVs stimulated a broad cytokine secretion from human macrophages. No statistical differences were observed between MVs from TB treatment.
